# Pharmacological treatment of hypertension and hyperlipidemia in Izhevsk, Russia

**DOI:** 10.1186/s12872-016-0300-9

**Published:** 2016-06-03

**Authors:** Marta Cybulsky, Sarah Cook, Anna V. Kontsevaya, Maxim Vasiljev, David A. Leon

**Affiliations:** McGill University, Montréal, Canada; Department of Non Communicable Disease Epidemiology, London School of Hygiene & Tropical Medicine, Keppel Street, London, WC1E 7HT UK; National Research Centre for Preventive Medicine, Moscow, Russia; Izhevsk State Medical Academy, Izhevsk, Russia; Arctic University of Norway, UiT, Tromsø, Norway

**Keywords:** Hypertension, Hyperlipidemia, Russia, Prevalence, Treatment, Antihypertensive agents, Anticholesterolemic agents

## Abstract

**Background:**

Cardiovascular disease (CVD) is the leading cause of death in Russia. Hypertension and hyperlipidemia are important risk factors for CVD that are modifiable by pharmacological treatment and life-style changes. We aimed to characterize the extent of the problem in a typical Russian city by examining the prevalence, treatment and control rates of hypertension and hyperlipidemia and investigating whether the specific pharmacological regimes used were comparable with guidelines from a country with much lower CVD rates.

**Methods:**

The Izhevsk Family Study II included a cross-sectional survey of a population sample of 1068 men, aged 25–60 years conducted in Izhevsk, Russia (2008–2009). Blood pressure and total cholesterol were measured and self-reported medication use was recorded by a clinician. We compared drug treatments with the Russian and Canadian treatment guidelines for hypertension and hyperlipidemia.

**Results:**

The prevalence of hypertension was 61 % (age-standardised prevalence 51 %), with 66 % of those with hypertension aware of their diagnosis and 50 % of those aware taking treatment. 17 % of those taking treatment achieved blood pressure control. The majority (59 %) of those taking treatment were not doing so regularly. Prevalence of hyperlipidemia was 45 % (age-standardised prevalence 40 %), however less than 2 % of those with hyperlipidemia were taking any treatment. Types of lipid-lowering and anti-hypertensive medications prescribed were broadly in line with Russian and Canadian guidelines.

**Conclusion:**

The prevalence of hypertension and hyperlipidemia is high in Izhevsk while the proportion of those treated and attaining treatment targets is very low. Prescribed medications were concurrent with those in Canada, but adherence is a major issue.

## Background

Cardiovascular disease (CVD) is currently the leading cause of death worldwide [1]. The situation is striking in Russia, where levels of CVD mortality are among the highest in the world particularly for men [2–4].

Over the past five decades, Western countries have enjoyed a decline in CVD morbidity and mortality [5, 6]. This trend can be attributed to improved medical treatment and to population-level changes in exposure to CVD risk factors [5–8]. Canada is an example of a country where cardiovascular health promotion has become a priority on the national agenda. Guidelines for physicians on the diagnosis and treatment of hypertension and hyperlipidemia have been implemented [9, 10]. Education programs on CVD prevention are in place, and resources for management of risk factors are accessible to patients and the public [9, 10].

The situation is different in Russia. The longest period of decline in CVD mortality for many decades only started in 2005/6 and is still ongoing. Moreover, concerted efforts to develop a modern national strategy and infrastructure to address CVD morbidity and mortality have only been prioritized over the past 10–12 years. In 2006, President Vladimir Putin launched the National Priority Project Health to improve primary care and disease prevention, and to facilitate accessibility to tertiary care and maternal and child health services [11]. In 2008, the priorities were expanded to include a reduction in CVD mortality [11]. In 2009, the Pharmaceutical 2020 concept was introduced to improve CVD detection and treatment by implementing patient registers, with the desired aim of introducing a more comprehensive drug insurance plan to cover prescription medications [11, 12]. In 2011, outpatient pharmaceutical coverage was expanded to include certain CVD drugs.

Hypertension and hyperlipidemia are key areas to investigate since these are modifiable risk factors for CVD which can be controlled with lifestyle change and pharmacological intervention [1]. Russia has published guidelines for the investigation and treatment of hypertension and hyperlipidemia that broadly follow the European guidelines [13]. These are mainly consistent with Canadian guidelines however very little is known about the specific types of medications prescribed and the frequency of use among individuals to whom they have been prescribed.

We investigated the prevalence and pharmacological treatment practices of hypertension and hyperlipidemia among working-age men in the city of Izhevsk, Russia, focusing on specific drugs prescribed, in order to assess how these compare to those recommended elsewhere. The fieldwork for this study occurred in 2008–9, after the start of the CVD mortality decline and the introduction of a range of national strategies. Although improvements may have occurred more recently, this study is one of only a very small number to look in detail at the profile of pharmacological treatments employed in Russia at any point in the past decade.

## Methods

### Study design

The data analysed were from the Izhevsk Family Study II (IFS-2), a cross-sectional study of 1,531 working-age men (25–60 years) conducted in Izhevsk, Russia. Izhevsk is a medium-sized industrial city situated in the Western Ural region. Participants were originally recruited as controls for a case-control study of the association between hazardous drinking and mortality (2003–05) derived from a 2002 list of city residents frequency matched by age to cases [14]. IFS-2 involved following up the control men in 2008–9.

IFS-2 had two parts. First, participants were interviewed face-to-face by trained interviewers at their own home, where data were collected on socio-demographic factors and health behaviours including questions on smoking status. Men were then invited to a health check performed by a physician at a clinic 2–3 weeks after the interview. There were 1,068 men who took part in this second stage. The distribution of socio-demographic and health behaviour variables were very similar between the men who attended the health check and those who did not. Only the data from the men who attended the health check were used in our analyses.

The health check involved a complete medical history collected using a doctor-administered questionnaire and a standard medical examination. Seated systolic blood pressure (SBP) and diastolic blood pressure (DBP) were measured three times using Omron (705 IT) electronic sphygmomanometers. Participants were asked if they were aware of whether their blood pressure was higher in one arm and if so this arm was used for measurement. The mean of the second and third readings were used to determine hypertension prevalence and control. Three measures of waist and hip circumference were taken, height measurements used the SECA Leicester Portable Height Measure, and weight measurements were taken using Tanita HS-1632 electronic weighing scales. Means of these values were used to calculate mean body mass index and mean waist to hip ratio.

A non-fasting blood sample was also collected during the health check on the same day as blood pressure measurement. Samples were transported to a local laboratory where they were spun and aliquoted within 12 h of venipuncture. Aliquots were stored at −80 °C and transported to the Lytech laboratory in Moscow, where lipid assays were performed. These included high-density lipoprotein cholesterol (HDL-C), triglycerides and total cholesterol (TC). Low-density lipoprotein cholesterol (LDL-C) level was estimated using the Friedwald equation.

The names of any medications taken, their prescribed dose, and frequency of use were self-reported by participants and recorded as text. This information was translated and coded by an English-speaking Russian cardiologist into their corresponding generic (English) names and each medication was classified according to primary function (e.g. anti-hypertensive or lipid-lowering).

We compared findings from Izhevsk with Canadian guidelines, which are similar to those from Western Europe. Since the implementation of the Canadian Hypertension Education Program (CHEP) in 1999, there have been marked increases in the diagnosis and treatment of hypertension in Canada [15].

### Hypertension measures

Standard definitions of hypertension used for comparative purposes in this paper were as given in the 2012 Canadian Hypertension Education Program [10]. Hypertension prevalence was defined as SBP ≥140 mmHg and/or DBP ≥90 mmHg and/or self-reported use of prescribed drugs for BP control at the time of the health check. The data used to determine awareness and treatment of hypertension were obtained from participants’ responses to the doctor-administered questionnaire. Hypertension awareness was defined as any self-reported prior diagnosis of hypertension by a doctor or nurse, amongst those defined as hypertensive. The following questions were used: ‘Have you ever had your blood pressure measured by a doctor or a nurse?’ and ‘Have you ever been told by a doctor or nurse that you had high blood pressure?’ Hypertension treated was defined as self-reported use of a medication prescribed by a doctor for the management of high BP daily or intermittently, amongst those defined as hypertension aware. The following questions were used: ‘Has a doctor prescribed drugs to control your blood pressure?’ and (if yes) ‘Do you take these drugs?’ Options for participants were ‘Daily’ or ‘Only sometimes when I feel unwell (coded as intermittently)’ and ‘Never’. Hypertension controlled was defined as mean SBP <140 mmHg and/or DBP <90 mmHg, amongst those who reported having been prescribed anti-hypertensives.

### Hyperlipidemia measures

Definitions of hyperlipidemia used for comparative purposes in this paper were adapted from the 2009 Canadian Cardiovascular Society Guidelines for the Diagnosis and Treatment of Dyslipidemia [9]. Hyperlipidemia prevalence was defined as LDL-C ≥3.5 mmol/L and/or TC/HDL-C ≥5 and/or use of prescribed lipid-lowering medications at the time of the health check. The self-reported prescribed lipid-lowering medications recorded during the health check were used to determine treatment of hyperlipidemia amongst those defined as hyperlipidemic. No questions were included related to self-reported awareness of hyperlipidemia. Hyperlipidemia controlled was defined as LDL-C <3.5 mmol/L and TC/HDL-C <5 amongst those defined as hyperlipidemia treated.

### Analyses

**Fig. 1:**
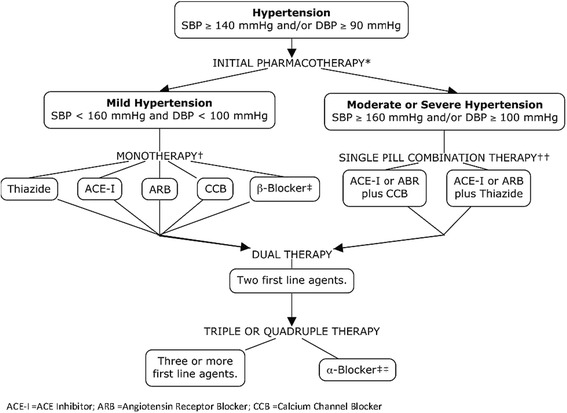
Anti-hypertensive Pharmacotherapy Treatment Guidelines in 2012 Canadian Hypertension Education Programme

The prescribed anti-hypertensive medications used by participants were compared with the treatment guidelines in the 2012 Canadian Hypertension Education Program [10]. Anti-hypertensive pharmacotherapy according to these guidelines is summarized in Fig. 1. Two analyses were done. In the first, all anti-hypertensive medications recorded were analyzed by frequency of prescription and whether or not they were currently prescribed in Canada. The second analysis investigated first-line pharmacotherapy judged by examining prescriptions among men only taking a single anti-hypertensive drug (including single pill combination drugs). Since no health records were available it was not possible to distinguish separately those who may have changed drugs treatments but this still allows us to distinguish preferential prescription of monotherapies.

The prescribed lipid-lowering medications used by participants were compared against the 2009 Canadian Cardiovascular Society Guidelines for the Diagnosis and Treatment of Dyslipidemia [9]. According to the guidelines, a statin should be initial pharmacotherapy for adults with hyperlipidemia without other compelling medical conditions. If statin therapy is not tolerated, LDL-C may be lowered using niacin, bile acid resins, cholesterol absorption blockers, and fibrates. Due to very low levels of hyperlipidemia treatment among study participants, only an assessment of whether or not the lipid-lowering medications were concurrent with those prescribed in Canada was performed.

Statistical analyses were performed using STATA 12 [16]. Cross-tabulations were performed to provide estimates of prevalence, awareness, treatment, and control of hypertension and hyperlipidemia. Prevalence of hypertension and hyperlipidemia were directly standardised by age using the 1976 European Standard Population so results could be compared with the ESSE-RF study carried out in nine regions of Russia between 2012 and 2013 [17].

## Results

A total of 1068 men attended the health check. The mean age was 48.1 years. The age distribution was skewed left, with more men in older age categories. The majority of men did not use medications of any kind (71 %). Most (97 %) reported ever having had their BP measured by a doctor or nurse. There were 669 men (62.6 %) who were current smokers. Mean body mass index was 26.3 (SD 4.4) and mean waist to hip ratio was 0.93 (SD 0.07). The prevalence of abdominal obesity defined as mean waist to hip ratio >0.9 was 64.8 %. Distribution of the sample by hypertension stage according to measured blood pressure (not accounting for use of anti-hypertensive medications) was 334 (31.5 %) of the sample had stage 1 hypertension (SBP 140–159 mm Hg or DBP 90–99 mm Hg) and 281 (26.5 %) of the sample had stage 2 hypertension (SBP ≥160 mm Hg or DBP ≥100 mm Hg).

### Hypertension

**Fig. 2:**
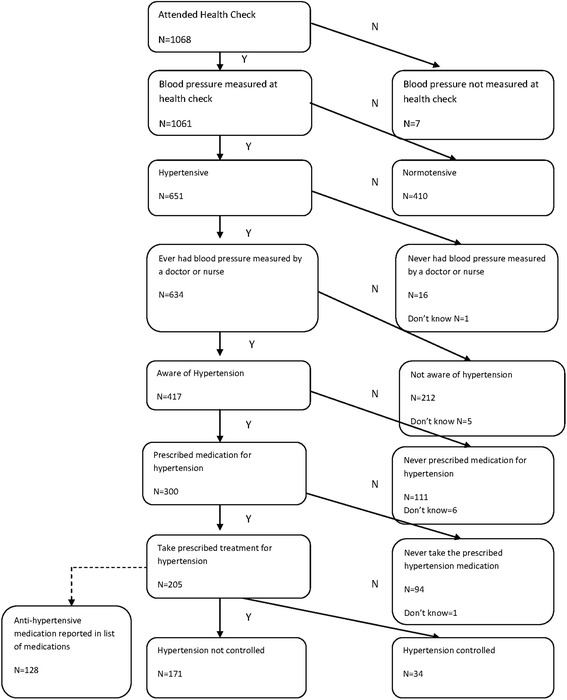
Number of men included and excluded in hypertension prevalence, aware, treated and controlled categories

The flowchart in Fig. 2 shows for hypertension how the men included in the study were classified according to information obtained about awareness, diagnosis, treatment and results at the health check. Of the 1068 men who underwent the health check, BP was successfully measured in 1061 men. The prevalence of hypertension was 61 % (651/1061). The age standardized prevalence of hypertension was 51 %.

Table 1 shows the distribution by age of hypertension prevalence, awareness, treatment, and control amongst those taking treatment for hypertension. Prevalence, awareness and treatment all increased with age, however control showed the opposite effect.

**Table 1:** Hypertension prevalence and percentage of those with hypertension who are aware, treated, and controlled by age

	Prevalence^a^			Awareness^b^			Treated^c^			Controlled^d^		
Age (years)	Total	*N*	%	Total	*N*	%	Total	*N*	%	Total	*N*	%
25–39	189	74	39.15	70	39	55.71	37	13	35.14	13	5	38.46
40–49	328	187	57.01	178	109	61.24	109	43	39.45	43	9	20.93
50–60	544	390	71.69	386	269	69.69	265	149	56.23	149	20	13.42
25–60	1061	651	61.36	634	417	65.77	411	205	49.88	205	34	16.59

^a^Hypertension prevalence: Mean of the second and third SBP readings ≥140 mmHg and/or DBP ≥90 mmHg and/or self-reported use of prescribed drugs for BP control

^b^Hypertension awareness: Any self-reported, prior diagnosis of hypertension by a doctor or nurse, amongst those defined as hypertension prevalent

^c^Hypertension treated: Self-reported use of a medication prescribed by a doctor for the management of high BP daily or intermittently, amongst those defined as hypertension aware excluding those who reported they did not know if they had been prescribed hypertension medication (*n* = 6)

^d^Hypertension controlled: Mean of the second and third SBP readings <140 mmHg and DBP <90 mmHg, amongst those defined as hypertension treated

Among those taking prescribed anti-hypertensive medication, less than half (41 %) (85/205) were taking treatment every day (Table 2).

**Table 2:** Frequency of taking hypertension treatment prescribed by a doctor by age

Age (years)	Takes treatment daily^a^		Takes treatment intermittently^b^		Treated total^c^	
	*N*	%	*N*	%	*N*	%
25–39	4	31	9	69	13	100
40–49	15	35	28	65	43	100
50–60	66	44	83	56	149	100
25–60	85	41	120	59	205	100

^a^Men who report taking hypertension treatment prescribed by a doctor every day, amongst those defined as hypertension treated

^b^Men who report taking hypertension treatment prescribed by a doctor intermittently (less than once daily), amongst those defined as hypertension treated

^c^Hypertension treated: Self-reported use of a prescription medication for the management of high BP daily or intermittently, amongst those defined as hypertension aware

### Types of anti-hypertensive medication

There were 128 men taking one or more prescribed anti-hypertensive medications according to the drugs named in the list of medications.

Table 3 shows the types of anti-hypertensive drugs used, the number of times each drug was reported, for all men and for men taking one drug only (monotherapy), and whether or not the drug is currently prescribed in Canada. Drugs have been arranged by class in the order of frequency by which they were prescribed in Izhevsk. ACE Inhibitors (ACE-I) were the most common drugs reported (43 % of prescriptions). The use of combination therapy was very low (11 %). A local medication (Reserpine) was recorded twice (<2 %). With the exception of the local medication, all the prescribed anti-hypertensive drugs taken by the men are also currently prescribed in Canada.

**Table 3:** Prescription frequency of anti-hypertensive medications by type for all men (*N* = 173 prescriptions) and for men taking monotherapy (*N* = 90 prescriptions)

All anti-hypertensive drugs (100 % of prescriptions)		Total	Monotherapy	Prescribed in Canada
		N	N	
		173	90	
Thiazide Diuretics (3.5 % of total prescriptions)	Indapamide	5	3	Yes
	Hydrochlorothiazide	1	--	Yes
	Total	6	3	
ACE-Inhibitors (42.8 % of total prescriptions)	Enalapril	58	36	Yes
	Lisinopril	7	5	Yes
	Perindopril	6	4	Yes
	Captopril	1	--	Yes
	Fosinopril	1	--	Yes
	Ramipril	1	--	Yes
	Total	74	45	
ARBs (0.6 % of total prescriptions)	Losartan	1	--	Yes
	Total	1	0	
Calcium-Channel Blockers (11.6 % of total prescriptions)	Nifedipine	8	2	Yes
	Verapamil	7	1	Yes
	Amilodipine	5	1	Yes
	Total	20	4	
β-Blockers (28.9 % of total prescriptions)	Metoprolol	32	13	Yes
	Bisoprolol	15	8	Yes
	Propranolol	2	--	Yes
	Carvediol	1	--	Yes
	Total	50	21	
Combination therapies (11.1 % of total prescriptions)				
ACE-Inhibitor & Diuretic (6.8 % of total prescriptions)	Perindopril & Indapamide	8	7	Yes
	Enalapril & Indapamide	1	1	No. Drugs prescribed separately.
	Enalapril & Hydrochlorothiazide	1	--	Yes
Beta-Blocker & Diuretic (6.2 % of total prescriptions)	Atenolol & Chlortalidone	9	8	Yes
	Total	19	16	
α-Blockers (0.6 % of total prescriptions)	Clonidine	1	1	Yes
	Total	1	1	
Local Medications (1.2 % of total prescriptions)	Reserpine (Rauwolfia alkaloid)	2	--	No. Drug not prescribed due to serious adverse side effects.
	Total	2	0	

Among men the 90 men on monotherapy (taking one anti-hypertensive drug only) half were taking an ACE-I. With the exception of one man taking an α-blocker, all men were taking a drug considered compatible with first-line anti-hypertensive therapy in Canada.

### Hyperlipidemia

**Fig. 3:**
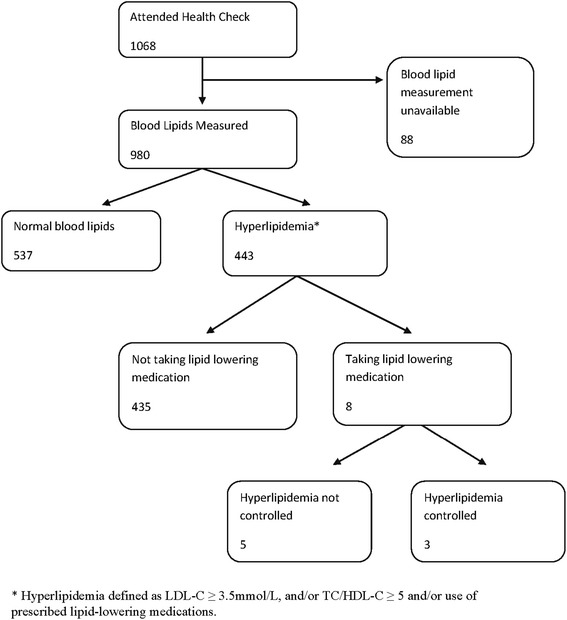
Number of men included and excluded in hyperlipidemia prevalence, treated and controlled categories

The flowchart in Fig. 3 shows inclusion criteria for hyperlipidaemia analyses. Of the 1068 men who underwent the health check, blood lipid measurements were available for 980 men (92 %). The prevalence of hyperlipidemia was 45 % (443/980). The age standardized prevalence was 40 %.

Amongst those classified as hyperlipidemia prevalent, less than 2 % (eight men) were taking lipid-lowering medications. Of those taking medications, 38 % (3/8) men achieved blood lipid control. Seven of these men were taking Simvastatin and one man was taking a lipid lowering drug which was not classified by name. Additionally there was one man who had no lipid measurement available who was taking Atorvastatin. Both Simvastatin and Atorvastatin are currently prescribed in Canada for first-line medical treatment of hyperlipidemia.

## Discussion

In this population-based study of working-age men in Izhevsk 2008–9, Russia, we found low rates of control of hypertension and that among those men prescribed anti-hypertensives very few took them regularly. Even more striking was the finding that very few people with hyperlipidemia (<2 %) were taking lipid-lowering medication. Nonetheless, the specific types of anti-hypertensive and lipid-lowering medications reported as being taken by participants were broadly consistent with those recommended in clinical guidelines in both Russia and a high income country (Canada) with much lower CVD mortality. Thus the high rate of CVD mortality observed in this Russian population is unlikely to be explained by the use of out-dated or ineffective pharmaceuticals. However the findings from this study indicate that the extent of pharmaceutical prescriptions and levels of adherence to treatment could be important factors contributing to high mortality from CVD in Russia.

The prevalence of hypertension in our study was 61 % (age-standardised prevalence 51 %). In the most recent study ESSE-RF performed in nine regions of the Russian Federation in 2012–2013 age-standardised prevalence of hypertension in men aged 25–64 years was 48.2 %, very similar to our findings [17]. However this is high compared with data from the Federal hypertension monitoring program (2003–2010) which showed the age-standardised prevalence of hypertension as 38 % in men although as the age range for this population is much wider than in our study (15–75 years) these results are not directly comparable with Izhevsk [18]. A study by Mozheyko et al. investigating control levels amongst hypertensive patients in the Yaroslavl region of Russia found at baseline, the level of blood pressure control amongst those taking anti-hypertensive treatment was 16.8 %. Control rates improved to 23.0 % at 1-year follow-up [19]. In the recent ESSE-RF study levels of control among men receiving treatment was higher (41.4 %) although overall levels of blood pressure control in all men with hypertension was 14.4 % [17]. In contrast in the nationally representative Canadian Health Measures Study (2007–9) prevalence of hypertension among adults aged 20–79 years old in Canada was estimated at 19.7 % while levels of treatment and control were notably higher compared with Russian studies with 79 % of those with hypertension taking regular treatment and 64.6 % of those with hypertension both treated and controlled [20]. Although prevalence estimates are not directly comparable since data from the Canadian Health Measures Survey were not age-standardised the difference in prevalence is so large it is unlikely to be completely explained by differences in age structure of the study populations.

Effective blood pressure control depends on patient adherence since target blood pressure can only be achieved and maintained with regular, daily therapy. However in our study approximately 60 % of men prescribed medication for hypertension did not take it on a daily basis. Our findings are consistent with a recent population-based study by Roberts et al. on hypertension treatment in countries of the former Soviet Union, which found that approximately three quarters of people taking anti-hypertensive medications were not taking them every day [21]. Studies of adherence in primary care settings shows low adherence even in patients at a specialised clinic with complicated hypertension [22]. All these studies suggest irregular use of hypertension medication is a major issue. Use of combination therapy may help with treatment adherence by simplifying treatment regimes when more than one drug is prescribed. The use of combination therapies has increased over the last decade in Canada, the US and Western Europe [23–25] however in our study, in keeping with other recent studies in Russia [19, 26], the use of combination therapies was relatively low.

To our knowledge, this is the first population study to investigate in detail type of prescribed medications used for treatment of hypertension in Russia. Our findings are consistent with studies from clinical settings that prescribed medical treatments for hypertension are modern and in keeping with medical guidelines from both Russia and Canada [22, 27, 28]. We believe this is the first study to investigate detailed treatment of hyperlipidemia in any setting in Russia. Age standardised prevalence of hyperlipidemia in our study from 2009 (40 %) was slightly lower than from the more recent ESSE-RF study from 2012 to 2013 (47.8 %) [29, 30] and the Canadian Health Measures Survey (45 % not age-standardised). This is high compared to the WHO MONICA study which surveyed participants in Novosibirsk and Moscow between 1992 and 1995 (age standardized range in men 8–16 %) although the threshold used for hyperlipidemia was higher (total cholesterol ≥6.5 mmol/L or use of lipid lowering medication) [31]. However there were substantial differences between Izhevsk and the Canadian Health Measures Study with regards the levels of pharmacological treatment for hyperlipidemia with less than 2 % of men with hyperlipidemia taking lipid lowering medications in Izhevsk compared to 24 % of people with hyperlipidemia in Canada [32]. Nonetheless, the medications taken were the same as those prescribed in Canada. It is worth noting that even though more people were on treatment for hyperlipidemia in the Canadian Health Measures Survey, the overall levels of treatment and of control in Canada were still low (19 % controlled) suggesting improvements could be made in both countries [32]. Levels of treatment in Russia for hyperlipidemia in the MONICA study (1992–95) showed a great deal of variation ranging by centre with 6–25 % of men (20–79 % women) with hyperlipidemia prescribed medications in Moscow and 0–48 % in Novosibirsk (0–39 % in women) [31]. Findings from our study are also supported by those from a recent large multicentre study of 18,273 patients visiting primary care facilities in Russia which found that half of those with hypercholesteremia were not prescribed treatment [33]. One possible explanation for the low treatment levels found in Izhevsk may stem from differences in preventative practices. A study by Pardell et al. [34] noted that routine blood cholesterol assessments were performed much less frequently by Russian primary care doctors in comparison to those in Western European countries. The reasons for this are not well understood. Cost may be a factor in explaining this since the routine measurement of blood lipids is an expensive clinical practice [34]. However insufficient statin treatment has been found even among patients on clinical registers under physician care with very high cardiovascular risk profiles and severe hypercholesterolemia [35, 36]. Although cost may be a factor in the low levels of statins prescribed, a wide range of cheap generic statins are currently available in Russia. We consider lack of knowledge and understanding of the importance of statin therapy by physicians and thus subsequently by patients likely to be a key factor in this. Clinical guidelines in Russia are very similar to Europe as they are translated and adapted from the guidelines of the European Society of Cardiology. The state of knowledge concerning the efficacy of statins in the Russian medical profession is underlined by a recent study of physician health in Russia which found that physicians themselves do not take statins even if they are at very high risk of cardiovascular disease (only 13 % of physicians who needed statin treatment actually took it) [37]. If physicians themselves do not think it is necessary to take statins themselves this decreases the chance of systematic prescription of statins to patients. Current physician attitudes to statins may be related to the fact that active promotion of statins by big pharmaceutical companies did not occur during the Soviet Union when drugs were still on patent. The reasons for the particularly low levels of treatment of hyperlipidemia and what can be done to improve this is a key area for further investigation.

It is important to note that the data we used and our analyses have several limitations.

Firstly our sample size was relatively small, particularly affecting the hyperlipidemia investigations with only 8 men taking lipid-lowering medications. Although we could not perform extensive investigations of lipid-lowering medications, the small number is an important finding in itself, as it reflects the extremely low levels of treatment. Our hyperlipidemia analyses were also limited by less extensive data collection compared to the hypertension data. No questions were asked in the study about awareness of hyperlipidemia, and whether or not participants had ever had their blood lipids measured. Such data would have been valuable to better assess primary care practices.

In addition our study did not collect information on the use of complementary and alternative medicine (CAM). CAM and other folk medical practices have been well-documented in Russia, and laws regulating these practices in Russia are limited [38]. It is therefore possible that the patients with hypertension and hyperlipidemia in our sample were using non-prescribed therapies, which may in part explain the low levels of conventional pharmacological treatment reported. Medications used were also self-reported by participants and therefore subject to measurement error. Blood pressure (measured three times) was only taken on one occasion in a clinic setting where it may be raised artificially due to medical environment and therefore the prevalence of hypertension may be over-estimated compared to clinically diagnosed hypertension that would require confirmation from a subsequent office blood pressure or use of a 24 h ambulatory device. In line with standard practice to minimize measurement error due to “white coat hypertension” only the second and third measurements were used in our analyses. Finally, there may have been some measurement error in the levels of hyperlipidemia since the study samples were non-fasting whereas the guidelines for identification were for fasting samples. This could have led to an overestimate of the true prevalence of hyperlipidemia. However the levels of treatment for hyperlipidemia were so low that the gap between prevalence and treatment is very unlikely to be explained by fasting status.

In terms of generalizability to the whole of Russia the study is very limited as it was conducted only in one city among working-age men and are therefore not applicable to women or the elderly. However, it could be regarded as a strength that it was conducted in a provincial city, and not one of the large metropolitan cities where standards of care might be expected to be the highest, but atypical of Russia as a whole.

It is also important to note our study was conducted in 2008–9. Since then, a national screening program for chronic diseases “Dispanserization” has been introduced in Russia. The situation therefore may have changed. More recent studies are needed investigating the impact of this program of CVD prevention and management. Findings from this study are important in understanding any changes in management which may have arisen from this initiative.

## Conclusions

Among a population-based sample of working-age men in Izhevsk the prevalence of both hypertension and hyperlipidemia was high and the levels of control of both conditions were extremely low. Among those with hypertension patient adherence to treatment was a particular issue whereas for hyperlipidemia hardly any men received treatment at all. Given both hypertension and hyperlipidemia are both important modifiable risk factors for CVD, which is the leading cause of death in Russia, this is a crucial public health issue.

## Abbreviations

ACE-I, ACE inhibitor; ARB, angiotension receptor blocker; BP, blood pressure; CCB, calcium channel blocker; CHEP, Canadian Hypertension Education Program; CVD, cardiovascular disease; DBP, diastolic blood pressure; HDL-C, high density lipoprotein cholesterol; IFS-2, Izhevsk Family Study 2; LDL-C, low density lipoprotein cholesterol; SBP, systolic blood pressure; TC, total cholesterol

## References

[1] World Health Organization (2013). A global brief on hypertension.

[2] Go AS, Mozaffarian D, Roger VL, Benjamin EJ, Berry JD, Borden WB, Bravata DM, Dai S, Ford ES, Fox CS (2013). Heart disease and stroke statistics—2013 update: a report from the American Heart Association. Circulation.

[3] Powles JW, Zatonski W, Hoorn S, Ezzati M (2005). The contribution of leading diseases and risk factors to excess losses of healthy life in Eastern Europe: burden of disease study. BMC Public Health.

[4] World Health Organisation. Global Status Report on noncommunicable disease 2014. Geneva; 2014. http://apps.who.int/iris/bitstream/10665/148114/1/9789241564854_eng.pdf?ua=1.

[5] Tunstall-Pedoe H (2012). The decline in coronary heart disease; did it fall or was it pushed?. BMJ.

[6] O’Flaherty M, Buchan I, Capewell S (2013). Contributions of treatment and lifestyle to declining CVD mortality: why have CVD mortality rates declined so much since the 1960s?. Heart.

[7] Mancia G, De Backer G, Dominiczak A, Cifkova R, Fagard R, Germano G, Grassi G, Heagerty AM, Kjeldsen SE, Laurent S (2007). 2007 Guidelines for the management of arterial hypertension: The Task Force for the Management of Arterial Hypertension of the European Society of Hypertension (ESH) and of the European Society of Cardiology (ESC). Eur Heart J.

[8] Ezzati M, Obermeyer Z, Tzoulaki I, Mayosi BM, Elliott P, Leon DA (2015). Contributions of risk factors and medical care to cardiovascular mortality trends. Nat Rev Cardiol.

[9] Genest J, McPherson R, Frohlich J, Anderson T, Campbell N, Carpentier A, Couture P, Dufour R, Fodor G, Francis GA (2009). 2009 Canadian Cardiovascular Society/Canadian guidelines for the diagnosis and treatment of dyslipidemia and prevention of cardiovascular disease in the adult – 2009 recommendations. Can J Cardiol.

[10] Daskalopoulou SS, Khan NA, Quinn RR, Ruzicka M, McKay DW, Hackam DG, Rabkin SW, Rabi DM, Gilbert RE, Padwal RS, Dawes M, et al., for the Canadian Hypertension Education Program. The 2012 Canadian Hypertension Education Program recommendations for blood pressure measurement, diagnosis, assessment of risk, and therapy. Can J Cardiol. 2012;28(3):270–87. 10.1016/j.cjca.2012.02.018 22595447

[11] Popovich L, Potapchik E, Shishkin S, Richardson E, Vacrouz A, Mathivet B. Russian Federation: Health system review. Health Syst Transit. 2011;13(7):1–190. 22455875

[12] Clark F (2013). Health and medicine under Putin. Lancet.

[13] Mancia G, Fagard R, Narkiewicz K, Redon J, Zanchetti A, Bohm M, Christiaens T, Cifkova R, De Backer G, Dominiczak A (2013). 2013 ESH/ESC guidelines for the management of arterial hypertension: The Task Force for the management of arterial hypertension of the European Society of Hypertension (ESH) and of the European Society of Cardiology (ESC). Eur Heart J.

[14] Leon DA, Saburova L, Tomkins S, Andreev E, Kiryanov N, McKee M, Shkolnikov VM (2007). Hazardous alcohol drinking and premature mortality in Russia: a population based case-control study. Lancet.

[15] Campbell NRC, Brant R, Johansen H, Walker RL, Wielgosz A, Onysko J, Gao R-N, Sambell C, Phillips S, McAlister FA (2009). Increases in antihypertensive prescriptions and reductions in cardiovascular events in Canada. Hypertension.

[16] StataCorp (2011). Stata Statistical Software: Release 12.

[17] Boytsov SA, Balanova YA, Shalnova SA, Deev AD, Artamonova GV, Gatagonova TM, Duplyakov DV, Efanov AY, Zhernakova YV, Konradi AO (2014). Arterial hypertension among individuals of 25–64 years old: prevalence, awareness, treatment and control. By the data from ECCD. Cardiovasc Ther Prev.

[18] Oganov RG, Timofeeva TN, Koltunova IE, Konstantinov VV, Balanova YA, Kapustina AV, Lelchuk IN, Shalnova SA, Deev AD (2011). Arterial hypertension epidemiology in Russia; the results of 2003–2010 federal monitoring. Cardiovasc Ther Prev.

[19] Mozheyko M, Eregin S, Vigdorchik A, Hughes D (2012). A cross-sectional survey of hypertension diagnosis and treatment practices among physicians in Yaroslavl Region, Russia. Adv Ther.

[20] McAlister FA, Wilkins K, Joffres M, Leenen FH, Fodor G, Gee M, Tremblay MS, Walker R, Johansen H, Campbell N (2011). Changes in the rates of awareness, treatment and control of hypertension in Canada over the past two decades. CMAJ.

[21] Roberts B, Stickley A, Balabanova D, McKee M (2012). Irregular treatment of hypertension in the former Soviet Union. J Epidemiol Community Health.

[22] Kontsevaya AV, Romanenko TS, Vygodin VA, Fitilev SB (2015). Evaluation of the regularity of antihypertensive drugs usage as a component of treatment adherence in outpatients of a specialized cardiology center. Ration Pharmacother Cardiol.

[23] Sever PS, Messerli FH (2011). Hypertension management 2011: optimal combination therapy. Eur Heart J.

[24] Gradman AH, Basile JN, Carter BL, Bakris GL (2010). Combination therapy in hypertension. J Am Soc Hypertens.

[25] Mallat SG, Itani HS, Tanios BY (2013). Current perspectives on combination therapy in the management of hypertension. Integr Blood Press Control.

[26] Konradi AO, Rotar OP, Korostovtseva LS, Ivanenko VV, Solntcev VN, Anokhin SB, Bart VA, Shlyakhto EV (2011). Prevalence of metabolic syndrome components in a population of bank employees from St. Petersburg, Russia. Metab Syndr Relat Disord.

[27] Kontsevaya AV, Romanenko TS, Vygodin VA, Fitilev SB (2015). Pharmacoepidemiology and the efficacy of antihypertensive treatment in real-life practice of the cardiology referral clinic. Ration Pharmacother Cardiol.

[28] Leonova MV, Belousov YB, Steinberg LL, Galitskyi AA, Belousov DY (2011). Pharmaco-epidemiology of arterial hypertension in Russia: the results of the pharmaco-epidemiological study PIFAGOR III. Russ J Cardiol.

[29] Boytsov SA, Balanova Yu A, Shalnova SA (2014). Hypertension among people aged 25–64: prevalence, awareness, treatment and control. According ESSE-RF study. Cardiovasc Ther Prev.

[30] Muromtseva GA, Kontsevaya AV, Konstantinov VV, Artamonova GV, Gatagonova TM, Duplyakov DV, Efanov AY, Zhernakova Y, Il'in VA, Konradi AO, et al. The prevalence of non-infectious disease risk factors in the Russian population in 2012–2013. The results of ECVD-RF. Cardiovasc Ther Prev. 2014;13(6):4–11. In Russian.

[31] Tolonen H, Keil U, Evans A, Hobbs MST, Jamrozik K, Thompson PL, Armstrong BK, Dobson A, Leeder S, Alexander H (2005). Prevalence, awareness and treatment of hypercholesterolaemia in 32 populations: results from the WHO MONICA project. Int J Epidemiol.

[32] Joffres M, Shields M, Tremblay MS, Connor Gorber S. Dyslipidemia prevalence, treatment, control and awareness in the canadian health measures survey. Can J Public Health. 2013;104(3):e252–7. 10.17269/cjph.104.3783 PMC6974261 23823891

[33] Akhmedzhanov NM, Nebieridze DV, Safaryan AS, Vygodin VA, Shuraev AY, Tkacheva ON, Lishuta AS (2015). Analysis of hypercholesterolemia prevalence in the outpatient practice (according to the ARGO study): part I. [Russian]. Ration Pharmacother Cardiol.

[34] Pardell H, Roure E, Drygas W, Morava E, Nussel E, Puska P, Uhanov M, Laaksonen M, Tresserras R, Salto E (2001). East-west differences in reported preventive practices. A comparative study of six European areas of the WHO-CINDI programme. Eur J Pub Health.

[35] Martsevich SY, Gaisenok OV, Tripkosh SG, Lukina YV, Zagrebel’nyy AV (2013). Real practice of statins use and its dependence on follow-up in the specialized medical centre in patients with high cardiovascular risk (according to the PROFILE register). [Russian]. Ration Pharmacother Cardiol.

[36] Ershova AI, Meshkov AN, Yakushin SS, Loukianov MM, Moseychuk KA, Martsevich SY, Zagrebelnyy AV, Vorobyev AN, Pereverzeva KG, Pravkina EA (2014). Diagnosis and treatment of patients with severe hypercholesterolemia in real outpatient practice (according to the RECVASA registry). Ration Pharmacother Cardiol.

[37] Drozdova LY, Martsevich SY, Voronina VP (2011). Evaluation of cardiovascular risk factors prevalence and efficacy of their correction in physicians. Estimation of physicians’ expertise in up-to-date clinical guidelines. Results of the “Physician’s health and education” study. Ration Pharmacother Cardiol.

[38] Stickley A, Koyanagi A, Richardson E, Roberts B, Balabanova D, McKee M. Prevalence and factors associated with the use of alternative (folk) medicine practitioners in 8 countries of the former Soviet Union. BMC Public Health. 2013;13(83). 10.1186/1472-6882-13-83 PMC3636035 23578173

